# Clinical evaluation of potential usefulness of serum lactate dehydrogenase (LDH) in 2019 novel coronavirus (COVID-19) pneumonia

**DOI:** 10.1186/s12931-020-01427-8

**Published:** 2020-07-06

**Authors:** Mei-ying Wu, Lin Yao, Yi Wang, Xin-yun Zhu, Xia-fang Wang, Pei-jun Tang, Cheng Chen

**Affiliations:** 1 Department of Pulmonary, The Fifth People’s Hospital of Suzhou, 10 Guangqian Road, Suzhou, 215000 China; 2 Department of Pulmonary, The Affiliated Infectious Hospital of Soochow University, 10 Guangqian Road, Suzhou, 215000 China; 3 Department of Respiratory and Critical Medicine, The First Affiliated Hospital of Soochow University, 899 Pinghai Road, Suzhou, 215000 China; 4 Institute of Respiratory Diseases, Soochow University, 708 Renmin Road, Suzhou, 215006 China

**Keywords:** COVID-19, Pneumonia, LDH, Chest CT

## Abstract

**Background:**

There was much evidence suggesting that the serum lactate dehydrogenase (LDH) levels reflect the extent of various pathophysiological processes. However, the current information about dynamic change of LDH in COVID-19 pneumonia has not been well investigated.

**Methods:**

Study was performed in 87 cases confirmed by COVID-19 infection. The serum LDH levels were determined at diagnosis and follow-up visits. The evaluation of clinical response to therapy was based on chest CT scan. We selected the value of LDH around the data of chest CT scan (− 1 ~ + 1 day).

**Results:**

At diagnosis, significant differences in LDH levels were found between non-severe and severe group (*P* < 0.05). It was demonstrated that increase or decrease of LDH was indicative of radiographic progress or improvement (*P* < 0.05). The time to LDH normalization (5.67 ± 0.55, days) was positively correlated with the time to radiographic absorption (5.57 ± 0.65 days, r = 0.53, *P* < 0.05). Applying the cut-off value of the increase in LDH has good specificity to predict disease progression.

**Conclusions:**

Serum LDH was validated for its potential usefulness as markers for evaluating clinical severity and monitoring treatment response in COVID-19 pneumonia.

## Introduction

The fast-growing outbreak of the 2019 novel coronavirus (COVID-19), which originated from Wuhan in central China, reached multiple continents in merely a month. Cross person-to-person transmission of this new virus can result in severe and fatal respiratory disease like acute respiratory distress syndrome (ARDS) in humans [1]. With the gradual recognition of COVID-19 pneumonia, professional consensus, guidelines and criteria were steadily established with the aim of preventing transmission and facilitating diagnosis and treatment.

Although COVID-19 pneumonia was considered as a low lethal disease, recognition of disease progression was an important decision point concerning intensive therapies [1, 2]. Accordingly, chest CT has been preferentially used as a stable marker of COVID-19 pneumonia, but clinical experiences have revealed the decrease of its availability in critical cases. Therefore, a more sensitive and specific disease-progression marker of COVID-19 pneumonia has been required.

Lactate dehydrogenase (LDH) is an enzyme implicated in the conversion of lactate to pyruvate in the cells of most body tissues and increased following tissue breakdown. Consequently, elevated serum LDH is present in numerous clinical conditions, such as hemolysis, cancer, severe infections and sepsis, liver diseases, hematologic malignancies, and many others. Nowadays, there was much evidence suggesting that the serum LDH levels serve as a non-specific indicator of cellular death in many diseases [3, 4]. However, the current information about dynamic change of LDH in COVID-19 pneumonia was very still.

Here, we hypothesized that certain LDH change might be correlated to the time course of COVID-19 pneumonia. We therefore measured LDH levels and related them to disease’s severity and status. It was aimed to establish serum LDH as a potential marker for monitoring treatment response in COVID-19 pneumonia.

## Patients and methods

### Patients

All 87 patients were admitted to The Fifth Hospital of Suzhou and diagnosed with COVID-19 pneumonia from Jan 10 to Feb 16, 2020. Diagnosis of COVID-19 infection in patients was made by positive test for viral RNA of respiratory secretions obtained by bronchoalveolar lavage, sputum, nasopharyngeal swab, or oropharyngeal swab. Demographic information, clinical characteristics (included medical history, severity and comorbidities) and chest CT scan results of each patient were obtained from the electronical medical record system of The Fifth hospital of Suzhou. Severity of COVID-19 was defined according to the diagnostic and treatment guideline for COVID-19 pneumonia issued by Chinese National Health Committee (Version 1–6). The study was approved by the Ethics Committee of our Institute of The Fifth People’s Hospital of Suzhou (2020–005).

### Treatment and evaluation

Clinical treatment and assessments were carried out by the diagnostic and treatment guideline for COVID-19 pneumonia issued by Chinese National Health Committee (Version 1–6). As multiple pulmonary CT scans provided reliable data, it was preferentially used as a gold standard of disease status.

### Serial determinations of LDH levels

The serum LDH was determined by VITROS® dry chemistry analyzer (Johnson, range from 313 to 618 U/L) [5, 6]. All operations are performed in strict accordance with the operating instructions. The serum LDH levels were investigated at diagnosis and at routine follow-up visits. The serial value of LDH was selected around the data of chest CT scan (− 1 ~ + 1 day).

### Definition

The time to LDH normalization referred to the time interval from increased LDH to normalized LDH. This CT image scoring system was an adaptation of a method previously used to describe idiopathic pulmonary fibrosis and severe acute respiratory syndrome (SARS) [7]. CT score was assigned to each lung and each lobe, based on the size of the infected area. The score ranged from 0 to 5, with score 0 for no infected area, 1 for less than 5%, 2 for 6–25%, 3 for 26–50%, 4 for 51–75%, and 5 for more than 75%. The peak progress on CT (PPC) was defined as the highest score of CT image, remarkable absorption on CT (RAC) was defined as reduced CT image score by 50% compared to PPC. The time to radiographic absorption (TRA) was established as the time interval from PPC to time point of beginning reduction in CT image score.

### Statistical analysis

The normality of all data was tested by Kolmogorov-Smirnov test. The levels of LDH were compared between non-severe patients and severe patients using *t* test. The values of LDH selected from the data of admission, PPC, and RAC were compared using paired-samples T test. The predictive ability of decrease or increase in LDH to corresponding radiographic absorption or progression was evaluated by X^2^ tests. Receiver operating characteristic (ROC) curves were calculated in order to select the cut-off level of an increase or decrease in serum LDH indicating progress or improvement of adjacent chest scan. The correlation of the time to LDH normalization and the TRA was analyzed by Pearson correlation. All tests were two-sided with a *P*-value of less than 0.05 being considered statistically significant.

## Results

### Demographics and clinical characteristics

Among those patients (Table 1), 47 were male and 40 were female. Their age was in the range of 1 to 70 years old, with median age of 44 years old, and 35.6% (31/87) of them were more than 50 years old. The patients were categorized into 77 non-severe and 10 severe cases on admission. Underlying comorbidity was found in 28 (32.2%) patients, including hypertension (6, 6.9%), diabetes (5, 5.7%), and chronic airway diseases patients (5, 5.7%).

**Table 1:** Baseline characteristics of infected patients

Baseline characteristics	n/value
Gender	
male	47
female	40
Age (years, median)	44
Smoking history	6
Underlying conditions	
Hypertension	6
Diabetes	5
Chronic airway diseases	5
Chronic kidney diseases	2
Hepatitis B	2
Hepatocellular carcinoma	1
Pregnancy	1
Clinical severity	
Non-severe	77
Severe	10

### The LDH level at admission

**Fig. 1:**
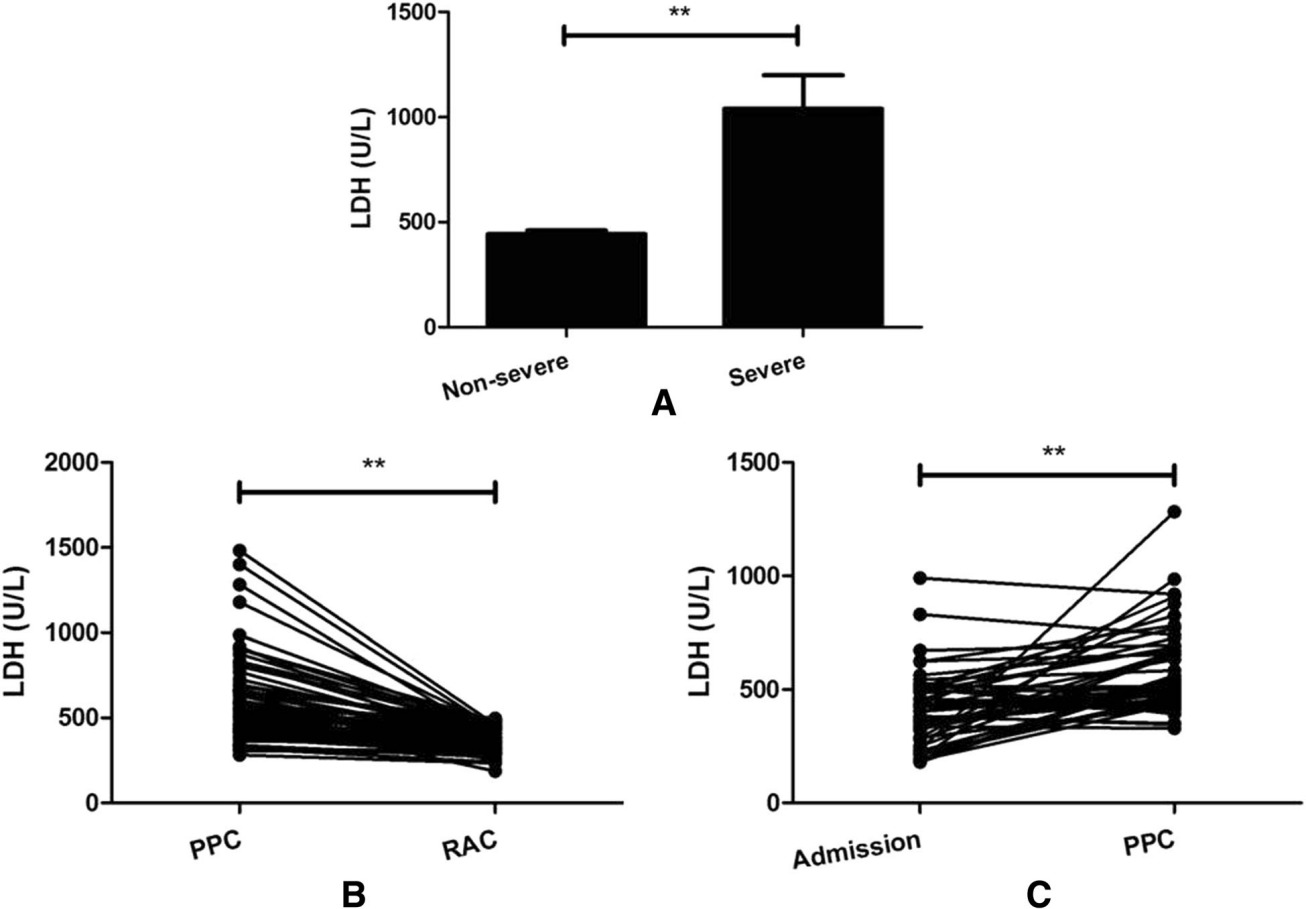
Lactate dehydrogenase (LDH) levels were determined in COVID-19 pneumonia patients during hospitalization. **a** differences in LDH levels were found between non-severe group and severe group (*P* < 0.05). **b, c** the values of LDH selected from the data of admission, peak progress on CT (PPC) and remarkable absorption on CT (RAC) were compared (*P* < 0.05). Notable, in some cases, the data of admission was under the peak course of disease

At admission, the LDH level in all patients was 495.1 ± 28.22 U/L (range 158–1482 U/L). The LDH level in non-severe patients amounted to 442.0 ± 17.47 U/L, the higher LDH levels were found in the severe group with a LDH level of 1040.0 ± 158.3 U/L (Fig. 1, *P* < 0.01).

### Correlation of fluctuation of LDH with radiographic change during hospitalization

Negative chest findings were revealed in 7 patients (7/87, 8.1%), repeated pulmonary CT still showed no abnormalities in these patients. Therefore, these features do not to allow the definition of COVID-19 pneumonia from others. Then, the remaining 80 patients were enrolled in following investigation.

During hospitalization, the majority of enrolling patients exhibited clinical progress. As multiple pulmonary CT scans and serial LDH determination provided reliable data, we investigated the correlation of serum LDH change with clinical status. As shown in Table 2, it was indicated that decrease or increase of LDH was indicative of corresponding radiographic improvement or progression (*P* < 0.05).

**Table 2:** Correlation of fluctuation of LDH with radiographic change during hospitalization in COVID-19 pneumonia patients

CT image	Progress	Absorption	*P* value
LDH			
Increase	54	30	< 0.01
Decrease	22	183	

As supported, compared to the initial LDH level (408.4 ± 23.77 U/L), the higher LDH levels were found in the following PPC with a level of 584.0 ± 27.14 U/L (Fig. 1, *P* < 0.05). When compared to the setting of PPC, the lower LDH levels were found in the following RAC (372.3 ± 8.25 U/L vs 578.9 ± 27.07 U/L, *P* < 0.05).

### LDH normalization and CT image improvement

**Fig. 2:**
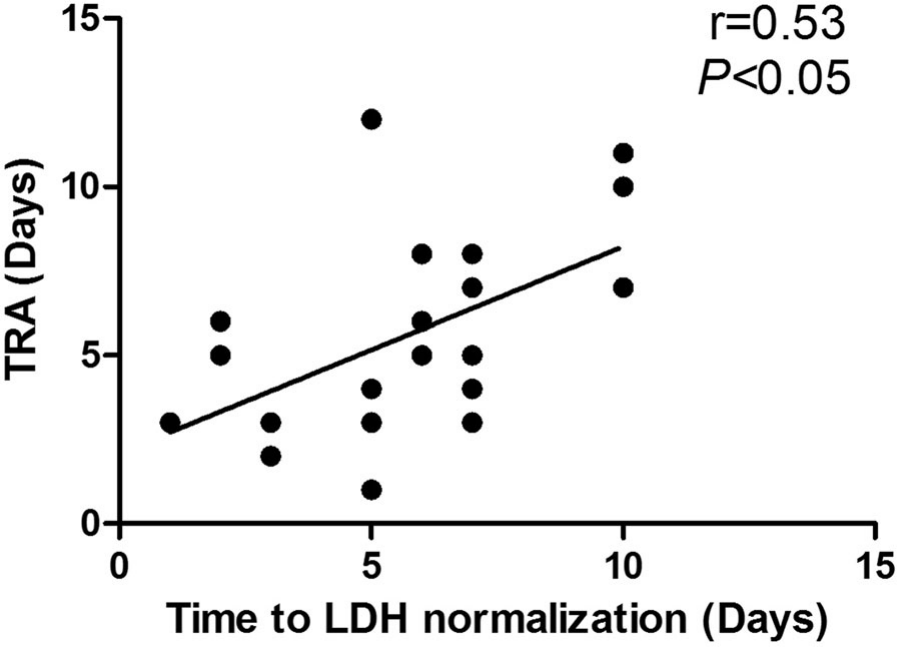
The correlation of the time to LDH normalization (days) with the time to radiographic absorption (TRA, days) was assessed by Pearson efficient

Of the 80 patients, 22 were noticed by increased LDH level above normal range (> 618 U/L). As shown in Fig. 2, base on the Pearson correlation coefficient, the data showed that the time to LDH normalization (5.67 ± 0.55, days) was positively correlated with time to radiographic absorption (5.57 ± 0.65 days, r = 0.53, *P* < 0.05).

### Predictive value of the LDH level to clinical overcome

**Fig. 3:**
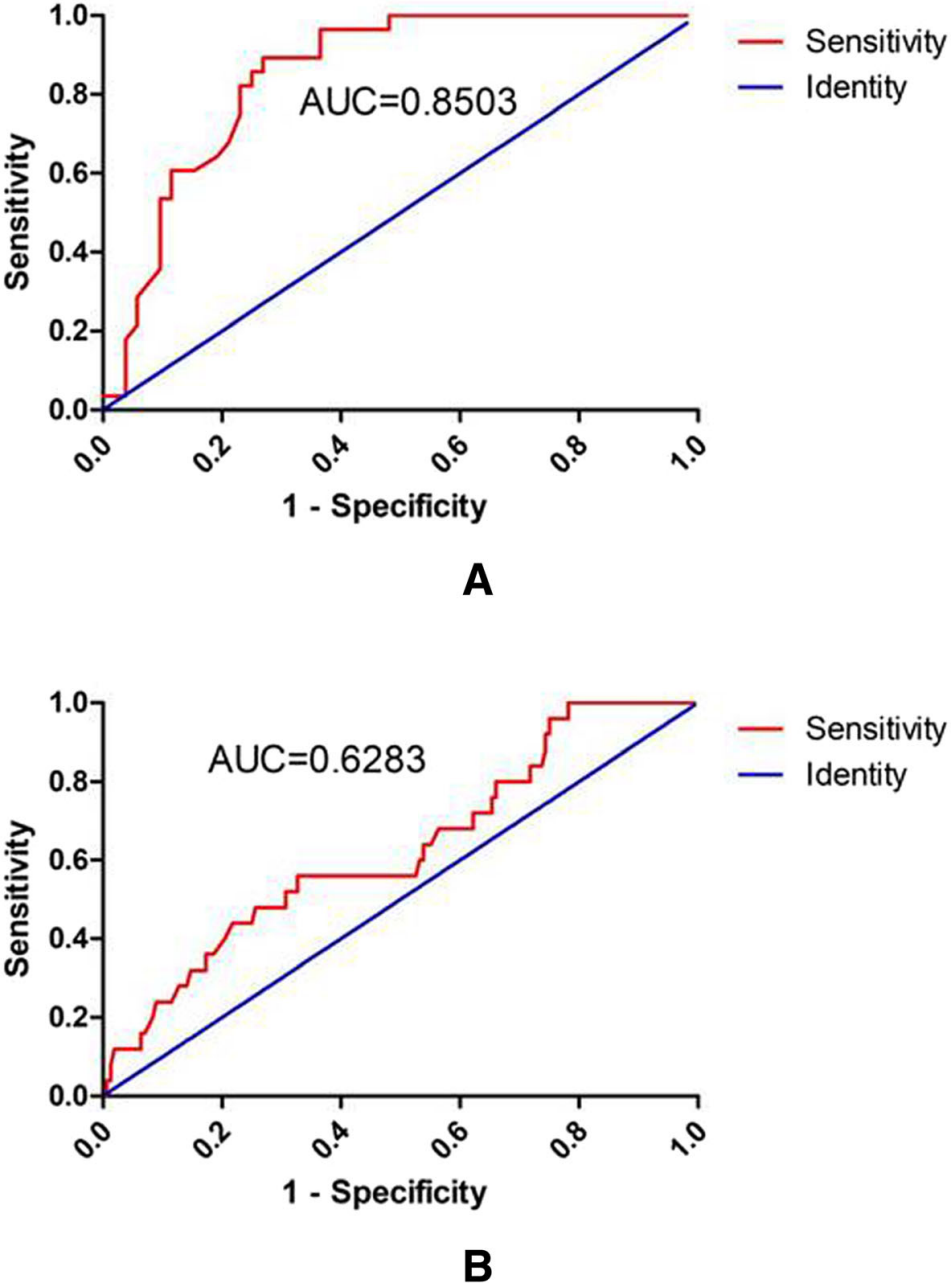
Predictive value of the LDH level to clinical overcome. **a** the optimal prognostic “cut-off value of LDH increase” predicting progress of the chest CT image was found to be 62.50 U/L, this criterion for sensitivity and specificity was found to be 73.1 and 89.3% respectively (AUC = 0.8503, *P* < 0.01). **b** The optimal prognostic “cut-off value of LDH decrease” predicting improvement of the chest CT image was found to be 48.50 U/L, the sensitivity and specificity was found to be 67.3 and 56.0% respectively (AUC = 0.6283 *P* = 0.04)

Following, to determine the optimal cut-off level for an increase or decrease in LDH indicating progress or improvement, ROC curves were calculated. Variations in adjacent LDH levels were computed as a function of the difference around the data of chest CT scan. As shown in Fig. 3, the optimal prognostic “cut-off value of LDH increase” predicting progress of the chest CT image was found to be 62.50 U/L. Applying the cut-off criterion, the sensitivity for disease progression was found to be 73.1% and the specificity was found to be 89.3%. The optimal prognostic “cut-off value of LDH decrease” predicting improvement of the chest CT image was found to be 48.5 U/L, its sensitivity and specificity was found to be 67.3 and 56.0%, respectively.

## Discussion

COVID-19 is an emerging viral illness that has rapidly transmitted throughout the world. From the early published literature, it has shown that the disease could induce symptoms including fever, dry cough, dyspnea and fatigue in infected patients [1, 2]. Clinically, some patients with COVID-19 pneumonia consistently demonstrated progress during the course of hospitalization, recognition of disease progression was an important decision point concerning intensive therapies.

The dynamic profile of laboratory findings has been tracked in patients with COVID-19 pneumonia. In the nonsurvivors, the neutrophil count, D-dimer, blood urea, and creatinine levels continued to increase, and the lymphocyte counts continued to decrease until death occurred. The risk factors indicated the importance of taking into account the disease severity, laboratory findings, chest imaging findings in practice [2, 8]. Under the circumstances, studies of the association between objective disease status and the laboratory findings may produce more interesting findings.

LDH can be released during tissue damage and is involved in various pathophysiological processes and serve as a non-specific indicator of cellular death in many diseases. A number of previous studies have shown that an elevated serum LDH is associated with a poor prognosis in malignancy [9–11]. In most scoring systems presented so far, prognostic variables including LDH were used as static variables determined at the time of diagnosis. The dynamics of the disease, however, may also be of great importance, especially when considering ‘decision points’ in treatment algorithms such as stem-cell transplantation [12].

For this reason, there was an urgent need to verify and update dynamic variables including LDH as the number of COVID-19 pneumonia accumulates. In the present study, serum LDH was validated for its potential usefulness as markers for evaluating clinical severity and monitoring treatment response in COVID-19 pneumonia. It was demonstrated that increase or decrease of LDH was indicative of radiographic progress or improvement. An increase in LDH by 62.5 U/L has an acceptable sensitivity and high specificity for a significantly higher probability of disease progression, when chest CT scan was employed to confirm the prediction. In support, during the whole observation period, normalization of serum LDH titer was consistently accurate in predicting treatment success in the patients.

It was known that kinds of disorders can raise LDH levels, such as infectious disease, heart failure, hypothyroidism and cancer. The inflammatory responses reflected the nonspecific responses to hypoxia, tissue injury, and necrosis, indicating a correlation between infectious cells, immune system and inflammatory response [13, 14]. Neutrophilia may be related to cytokine storm induced by virus invasion, coagulation activation could have been related to sustained inflammatory response [15]. In this study, complications of COVID-19 pneumonia included respiratory failure and liver injury. It is believed that the complication was signs of progression of disease, in turn produced influence on disease.

There are several limitations to our study. Firstly, almost all patients may have received medical intervention (antimicrobial therapy, fluid administration, mechanical ventilation, or steroid therapy), which may affect serum LDH titer. In addition, there may have been a selection bias in one medical institution. Moreover, a decrease in LDH by cut-off value has a poor sensitivity and specificity to predicting of image improvement, which might due to secondary organ dysfunction in late-term of diseases, not the direct the effect of pulmonary abnormality.

Taken together, our data suggested that LDH is a potentially useful follow-up parameter in COVID-19 pneumonia, which might assist in recognition of disease progression and thus help in risk stratification and early intervention.

## Data Availability

All data generated during this study are included in this published article.

## Abbreviations

ARDS: Acute respiratory distress syndrome
COVID-19: 2019 Novel Coronavirus
LDH: Lactate dehydrogenase
PPC: Peak progress on CT
RAC: Remarkable absorption on CT
ROC: Receiver operating characteristic
